# Effects of Wnt3A and mechanical load on cartilage chondrocyte homeostasis

**DOI:** 10.1186/ar3536

**Published:** 2011-12-09

**Authors:** Rhian S Thomas, Alan R Clarke, Victor C Duance, Emma J Blain

**Affiliations:** 1 Welsh School of Pharmacy, Redwood Building, Cardiff University, King Edward VII Avenue, Cardiff, CF10 3NB, UK; 2 Arthritis Research UK Biomechanics and Bioengineering Centre, School of Biosciences, Cardiff University, Museum Avenue, Cardiff, CF10 3AX, UK; 3 Division of Pathophysiology and Repair, School of Biosciences, Cardiff University, Museum Avenue, Cardiff, CF10 3AX, UK

## Abstract

**Introduction:**

Articular cartilage functions in withstanding mechanical loads and provides a lubricating surface for frictionless movement of joints. Osteoarthritis, characterised by cartilage degeneration, develops due to the progressive erosion of structural integrity and eventual loss of functional performance. Osteoarthritis is a multi-factorial disorder; two important risk factors are abnormal mechanical load and genetic predisposition. A single nucleotide polymorphism analysis demonstrated an association of hip osteoarthritis with an Arg324Gly substitution mutation in *FrzB*, a Wnt antagonist. The purpose of this study was two-fold: to assess whether mechanical stimulation modulates β-catenin signalling and catabolic gene expression in articular chondrocytes, and further to investigate whether there is an interplay of mechanical load and Wnt signalling in mediating a catabolic response.

**Methods:**

Chondrocytes were pre-stimulated with recombinant Wnt3A for 24 hours prior to the application of tensile strain (7.5%, 1 Hz) for 30 minutes. Activation of Wnt signalling, via β-catenin nuclear translocation and downstream effects including the transcriptional activation of *c-jun*, *c-fos *and *Lef1*, markers of chondrocyte phenotype (type II collagen *(col2a1)*, aggrecan *(acan), SOX9*) and catabolic genes (*MMP3, MMP13, ADAMTS-4, ADAMTS-5*) were assessed.

**Results:**

Physiological tensile strain induced *col2a1*, *acan *and *SOX9 *transcription. Load-induced *acan *and *SOX9 *expression were repressed in the presence of Wnt3A. Load induced partial β-catenin nuclear translocation; there was an additive effect of load and Wnt3A on β-catenin distribution, with both extensive localisation in the nucleus and cytoplasm. Immediate early response (*c-jun*) and catabolic genes (*MMP3, ADAMTS-4*) were up-regulated in Wnt3A stimulated chondrocytes. With load and Wnt3A there was an additive up-regulation of *c-fos*, *MMP3 *and *ADAMTS-4 *transcription, whereas there was a synergistic interplay on *c-jun*, *Lef1 *and *ADAMTS-5 *transcription.

**Conclusion:**

Our data suggest that load and Wnt, in combination, can repress transcription of chondrocyte matrix genes, whilst enhancing expression of catabolic mediators. Future studies will investigate the respective roles of abnormal loading and genetic predisposition in mediating cartilage degeneration.

## Introduction

Articular cartilage functions in withstanding mechanical loads by dissipating applied loads across the joint surface; it also provides frictionless movement of joints. The biomechanical integrity of the tissue is dictated by the composition of the extracellular matrix (ECM); the ability of cartilage to undergo reversible deformation is attributed to its unique ECM architecture. Cartilage ECM comprises a highly hydrated network of collagen fibrils (types II, IX and XI) embedded in a gel of negatively charged proteoglycans which together provide the tissue with tensile strength to resist compressive loads. Mechanical load is a potent anabolic regulator of cartilage ECM homeostasis; physiological loads up-regulate expression of type II collagen and the major proteoglycan aggrecan [1,2]. However, abnormal loading induced by joint misalignment, trauma or zero gravity inhibits ECM synthesis promoting catabolism via induction of proteolytic enzymes including collagen-degrading matrix metalloproteinases (MMPs) and the aggrecan-degrading aggrecanases (ADAMTSs) [1,3].

Osteoarthritis (OA) is a degenerative joint disease characterised by articular cartilage degradation, subchondral bone remodelling and osteophyte formation. The progressive erosion of structural integrity and eventual loss of functional performance is mediated by the MMPs, ADAMTSs and other matrix proteases. OA is a multi-factorial disorder - age, genetics and mechanical load are all contributing factors; the underlying molecular mechanisms are still largely unknown. Two separate genome-wide scans for familial OA susceptibility identified a locus on chromosome 2q which mapped to the gene *FrzB *[4]. Single nucleotide polymorphism (SNP) analysis demonstrated an association of hip OA with a functional SNP resulting in an Arg324Gly substitution in the encoded secreted frizzled-related protein 3 (sFRP3), a Wnt antagonist [4]. The substitution mutation in sFRP3 reduced Wnt inhibitory activity *in vitro *[4]. Wnt/β-catenin signalling components are essential for regulating cartilage development and chondrocyte function [5-7], and a number of Wnt and Wnt-associated proteins are elevated in murine and in human OA tissues [5,8]. Continuous activation of the canonical Wnt/β-catenin pathway can elicit matrix degradation [6]; in chondrocyte cultures, use of either Wnt3A conditioned media or forced expression of constitutive-active β-catenin stimulated transcription of MMP3, MMP13, ADAMTS-4 and ADAMTS-5 and increased proteoglycan loss [5,9].

More recently, *Col2a1-CreER^T2^; *β-*catenin^fx(Ex3)/wt ^*(β-catenin cAct) mice have been generated [9]; deletion of exon 3 containing critical GSK-3β phosphorylation sites results in the production of β-catenin which is resistant to GSK-3β phosphorylation and resultant ubiquitination. With age (< 8 months), there was a progressive loss of articular cartilage, development of fissures and osteophyte formation in the knee joints of β-catenin cAct mice.

Altered mechanical load is a major risk factor for OA [10,11], and excessive or abnormal joint loading patterns can initiate cartilage pathology [3,12]. Interestingly, in weight-bearing areas of the β-catenin cAct mice the articular cartilage surface was missing [9]. *FrzB *-/- mice, although devoid of any overt developmental abnormalities, are more susceptible to chemically-induced OA [13]. In addition, traumatic injury (by cutting) to human cartilage explants down-regulated *FrzB *and increased expression of canonical Wnt target genes [14].

Collectively, these studies raise the question as to the involvement of mechanical load in β-catenin-mediated cartilage degradation; to our knowledge this has not previously been reported. Therefore, the purpose of this study was two-fold: to assess whether mechanical stimulation modulates β-catenin signalling and catabolic gene expression in articular chondrocytes and, further, to investigate whether mechanical load and canonical Wnt signalling are intimately involved in mediating a catabolic response.

## Materials and methods

All chemicals were obtained from Sigma (Poole, UK) unless otherwise stated and were of analytical grade or above. Culture medium consisted of Dulbeccos Modified Eagle's Medium/Hams F12 (DMEM/F12 (1:1); GIBCO, Paisley, UK) supplemented with 100 μg/ml penicillin, 100 U/ml streptomycin and 1× insulin-transferrin-sodium selenite (1× ITS); the presence of ITS maintains the chondrocyte phenotype [15].

### Isolation of primary chondrocytes

Primary chondrocytes were isolated from the *metacarpophalyngeal *joint of seven-day-old bovine calves within six hours of slaughter as described previously [16]; ethical approval was not required for bovine tissue collection. Chondrocytes were plated at a high density of 4 × 10^6 ^cells per well in a six-well flat-bottomed pronectin coated plate (Bio-Flex culture plates; Dunn Laborotechnik, Asbach, Germany). Following isolation, cells were stabilised for 48 hours at 37°C in serum-free media containing 1× ITS.

### Application of mechanical load and Wnt3A

Chondrocytes were pre-treated with recombinant Wnt3A (150 ng/ml; Peprotech EC Ltd, London, UK) for 24 hours prior to the presence or absence of a physiological tensile strain (7.5% elongation, 1Hz, 30 minutes), which was applied using the Flexcell FX-3000 system (Flexcell International Corp, Hillsborough, NC, USA) [17,18]. As a control, cells were cultured equivalently in all steps but were devoid of Wnt3A and/or tensile strain. After application of strain, cells were either processed immediately or four hours post-load (henceforth referred to as immediate or recovery respectively).

### Analysis of gene expression using quantitative PCR

*C*ells were lysed in Trizol™ reagent (Invitrogen, Paisley, UK) and stored at -80°C prior to RNA extraction. Total RNA was extracted and complementary DNA generated as described previously [19]. Real-time PCR was carried out on Mx3000^® ^QPCR System (Stratagene, Leicester, UK). A real-time qPCR assay, based on SYBR Green^® ^detection, was designed for the transcriptional profiling of *c-fos*, *c-jun*, *Lef1, col2a1, acan, SOX9, MMP3, MMP13, ADAMTS-4 *and *ADAMTS-5 *in the cDNA samples using primers designed to the open reading frame of these bovine target genes [19-21] (for unpublished primers refer to Table 1). Data were normalised to *18s *rRNA levels [22] as treatments did not affect the expression of this gene. Reactions were carried out at an annealing temperature of 60°C unless indicated otherwise. Relative quantification was calculated using the 2^-ΔΔCT ^method as described previously [23], using the untreated controls as a reference group to quantify relative changes in target gene expression. The data are presented as fold change in expression normalised to an endogenous reference gene *18s *and relative to the untreated cDNA samples.

**Table 1:** Primer sequences of bovine genes of interest

Gene name	Primer sequence	T_m _(°C)	Product size (bp)
*Lef1*	For 5'-tcagcctgtgtatcccatca-3' Rev 5'-tgaggcttcacgtgcattag-3'	60	219
*c-fos*	For 5'-cggctttgcagacagagattg-3' Rev 5'-gggtgaaggcctcctcagatt-3'	60	192
*c-jun*	For 5'-taaactaagcccacgcgaag-3' Rev 5'-ctcagactggaggaacgagg-3'	60	100

### Analysis of protein expression using Western blotting

Cells were lysed in 500 μl 0.9% (v/v) Triton X-100^® ^containing a cocktail of phosphatase (inhibitor type I) and protease inhibitors (Calbiochem, Nottingham, UK). Equivalent amounts of protein extract (20 μg total protein determined using the BCA assay (Pierce, Cramlington, UK)) were separated on a 10% SDS-PAGE gel, transferred onto polyvinylidene fluoride (PVDF) membranes (Millipore, Consett, UK) and blocked in 1 × PBS (phosphate-buffered saline) containing 3% (w/v) skimmed milk powder. Membranes were probed overnight at 4°C with a rabbit polyclonal antibody which recognises both the phosphorylated and non-phosphorylated forms of β-catenin (1:1,000 dilution - clone 6F9), washed extensively with PBS containing 0.05% (v/v) Tween 20^® ^(PBS-T) and incubated with an affinity purified anti-mouse HRP-conjugated antibody (1:10,000 dilution) for 60 minutes at room temperature. Western blots were processed, scanned and analysed by densitometry as previously described [16]. Equivalent protein loading was confirmed by running Western blots (5 μg total protein) and probing with a monoclonal antibody which recognises the housekeeping protein GAPDH (1:1,000 dilution - clone GAPDH-71.1) in conjunction with goat anti-mouse IgM HRP-conjugated antibody (1:10,000 dilution - Abcam, Cambridge, UK). β-catenin protein levels are normalised to GAPDH and data are expressed as percentages of the untreated control.

### β-*catenin localisation using immunofluorescence and confocal microscopy*

Post-loading, chondrocytes were fixed with 2% (w/v) paraformaldehyde (in PBS) at room temperature for immunolocalisation studies. The Bio-Flex™ membrane was excised into nine squares (1 cm × 1 cm), chondrocytes permeabilised in 5% (v/v) Triton-X100 (in PBS) and blocked with 2% (v/v) goat serum for 60 minutes. Cells were incubated with mouse anti-β-catenin antibody (clone 6F9; 1:500 dilution), overnight, at 4°C, in a humidified chamber. After removal of primary antibody by repeated washes with PBS-T, cells were incubated with goat anti-mouse fluorescein isothiocyanate (FITC) secondary antibody (1: 64 dilution) for one hour each with repeated washes in PBS-T to remove residual antibody. Finally, the cells were mounted in VectaShield™ containing 1.5 μg/ml DAPI (Vector Laboratories, Peterborough, UK) and visualised using a confocal microscope (TCS SP2 AOBS Leica, Milton Keynes, UK) as previously described [16]. Representative cells were scanned using a 63× oil immersion objective with appropriate excitation and emission settings for FITC and DAPI. Maximum intensity 3D-reconstructions were prepared (Leica Confocal Software). For each experimental repeat, one excised membrane square was processed per well from three randomly selected wells per treatment group; between 4 and 6 cells were randomly imaged per membrane square; therefore, a total of between 36 and 54 cells were evaluated per experimental parameter.

### Statistical analysis

Data are presented as mean ± standard error mean (*n *= 6). Experiments were performed on three independent cell preparations, with between four and six legs accounting for each cell preparation; representative data are shown. Data were tested for normality (Anderson-Darling) and equal variance prior to parametric analyses (Minitab); where data were not normal and/or of equal variance, log transformations were performed prior to statistical analysis using a two-way ANOVA and Tukey's *post hoc *test. Differences were considered significant at *P *< 0.05.

## Results

### Wnt3A and tensile strain modulate β-catenin localisation in articular chondrocytes

**Figure 1:**
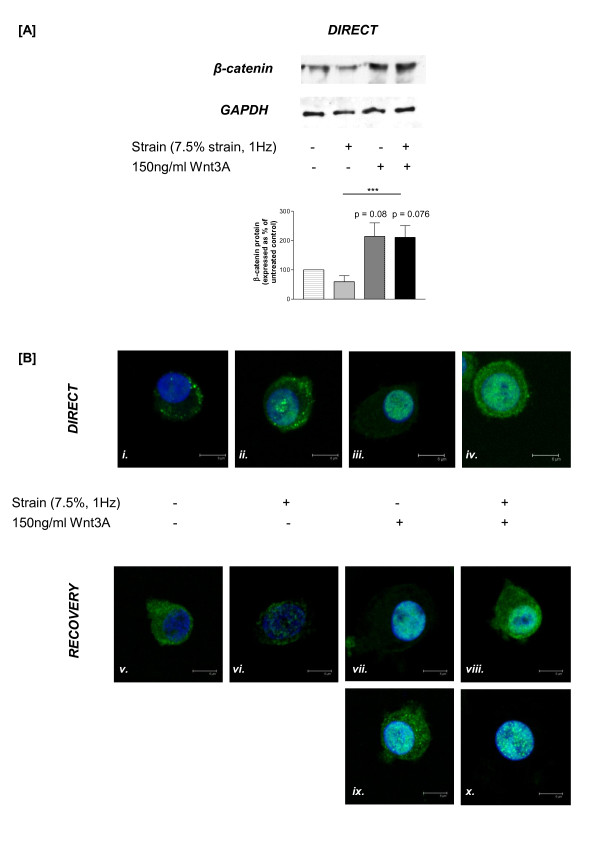
**β-catenin levels in chondrocytes subjected to tensile strain, separately or in combination with Wnt3A**. Untreated chondrocytes served as controls. **(A) **Representative Western blot depicting β-catenin protein expression detected in cells processed immediately (*direct*); equivalent protein levels were confirmed by probing for the housekeeping protein GAPDH. Densitometric data are presented as β-catenin levels expressed as a percentage of the untreated controls after normalisation to GAPDH; statistical analysis was performed on Western blots from three independent experiments. **(B) **β-catenin localisation, immediately following loading (i - iv) or after a four-hour recovery period (v - x) as detected using confocal microscopy. β-catenin was detected using a FITC-conjugated secondary antibody (green) and nuclei counterstained with DAPI (blue): representative 3D-reconstructions are presented (scale bar = 6 μm).

Mechano-regulation of β-catenin was assessed by quantifying protein levels (Figure 1A) and localisation of β-catenin within articular chondrocytes (Figure 1B). Following 30 minutes of tensile strain β-catenin expression levels were comparable to those in the untreated cells (*P *= 0.196; Figure 1A)). However, Wnt3A, in the presence (*P *= 0.076) or absence of tensile strain (*P *= 0.08) increased the amount of β-catenin protein detected (Figure 1A); levels did not quite reach significance due to blot to blot variability but the trends reported were consistent across the three independent experiments. Levels of the housekeeping protein GAPDH remained unaltered confirming equivalent protein loading. Similar trends were also observed in the cells following a recovery period of four hours post-cessation of strain (data not shown). In untreated cells, β-catenin was cytoplasmic in distribution (Figure 1B-i), and as expected, β-catenin was localised almost exclusively to the nucleus in response to Wnt3A stimulation (Figure 1B-iii). In contrast, partial nuclear translocation was observed in cells subjected to strain (Figure 1B-ii). In cells co-stimulated with tensile strain and Wnt3A, β-catenin was predominantly nuclear but there was also extensive labelling in the cytoplasm (Figure 1B-iv). Following the recovery period, β-catenin was cytoplasmic in distribution in both the untreated cells (Figure 1B-v) and those subjected to strain only (Figure 1B-vi). However, two different localisation profiles were observed in Wnt3A-treated cells, in the presence or absence of tensile strain. Approximately half of the cells in any particular field of view were comparable with the distribution observed at the earlier time point (compare Figures 1B-vii, viii with 1B-iii, iv). In the remainder of the Wnt3A treated cells, β-catenin expression was observed in both the nucleus and throughout the cytoplasm (Figure 1B-ix); whereas, in the cells co-stimulated with Wnt3A and strain, β-catenin was almost completely nuclear in its distribution (Figure 1B-x), comparable with the localisation observed in Wnt3A treated cells at the earlier time point (Figure 1B-iii). Negative controls, omitting the primary antibody, conducted in parallel were devoid of fluorescent signal (data not shown).

### Load-induced transcription of *c-fos*, *c-jun *and *Lef1 *is further regulated by Wnt3A

**Figure 2:**
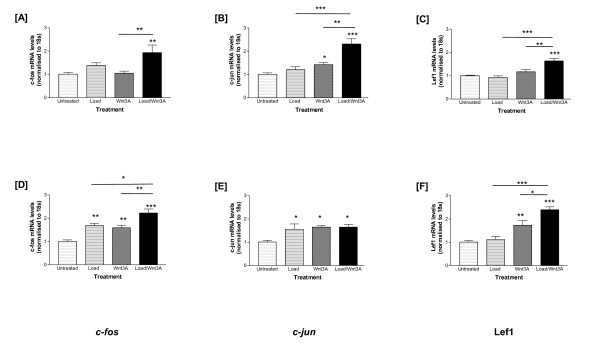
**Effects of tensile strain and Wnt3A, separately or combined, on chondrocyte early response gene transcription**. Untreated chondrocytes served as controls. **(A) ***c-fos*, **(B) ***c-jun *and **(C) ***Lef1 *gene expression levels analysed immediately or **(D)**, **(E) **and **(F) **following a four-hour recovery period respectively. Gene expression levels were assessed using quantitative PCR, normalised to the housekeeping gene 18s and relative to the untreated cells. Data are presented as mean ± S.E.M. (*n *= 6; *P < 0.05, **P < 0.01, ***P < 0.001).

mRNA levels of the immediate early genes *c-fos *(Figure 2A, D) and *c-jun *(Figure 2B, E) and the transcription factor *Lef1 *(Figure 2C, F) were assessed. In cells processed immediately, tensile strain had no effect on *c-fos *(Figure 2A) and *c-jun *transcription (Figure 2B). However, a synergistic induction of *c-fos *(1.9-fold; *P *< 0.01 compared to Wnt3A) and *c-jun *mRNAs (2.3-fold: *P *< 0.001 compared to untreated cells; *P *< 0.01 compared to Wnt3A) were observed in Wnt3A-treated cells subjected to strain (Figure 2A, B). Similarly, expression of the transcription factor *Lef1 *was not responsive to mechanical stimulation (Figure 2C), but tensile strain synergistically induced *Lef1 *transcription in Wnt3A-stimulated cells (1.65-fold: *P *< 0.001).

After recovery, elevated mRNA levels of *c-fos *(1.65-fold: *P *< 0.01, Figure 2D) and *c-jun *(1.5-fold: *P *< 0.05, Figure 2E) were observed in cells subjected to tensile strain. An additive effect of Wnt3A and tensile strain was observed on *c-fos *transcription after recovery (2.2-fold; *P *< 0.01 compared to Wnt3A). In contrast, *c-jun *mRNA levels were significantly elevated in cells irrespective of treatment. Wnt3A treatment independently increased *Lef1 *mRNA levels after recovery (1.7-fold; *P *< 0.01, Figure 2F), and the synergistic induction of *Lef1 *transcription remained in cells post-cessation of load (relative to untreated 2.4-fold: *P *< 0.001 or Wnt3A *P *< 0.05).

### Load-induced transcription of matrix synthesis genes is inhibited by Wnt3A

**Figure 3:**
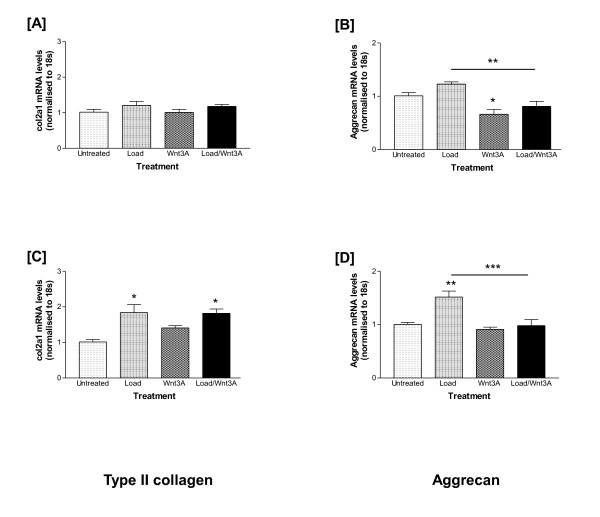
**Effects of tensile strain and Wnt3A, separately or combined, on chondrocyte marker gene expression**. Untreated chondrocytes served as controls. **(A) **type II collagen (*col2a1*) and **(B) **aggrecan (*acan*) gene expression levels analysed immediately or **(C) **and **(D) **following a four-hour recovery period respectively (refer to Figure 2 for calculations and statistical significance).

Markers of the chondrocyte phenotype, that is, type II collagen (*col2a1*; Figure 3A, C), aggrecan (*acan*; Figure 3B, D) and *SOX9 *(data not shown) were measured. Expression levels of *col2a1 *(Figure 3A) and *acan *(Figure 3B) did not alter in response to tensile strain in cells analysed immediately. Wnt3A stimulation did not affect *col2a1 *transcription, but reduced *acan *transcription directly (1.5-fold; *P *< 0.05) and in cells subjected to tensile strain (2-fold; *P *< 0.01).

After recovery, tensile strain induced *col2a1 *(1.8-fold; *P *< 0.01, Figure 3C) and *acan *(1.5-fold; *P *< 0.01, Figure 3D) transcription. Wnt3A had no effect on the transcription of chondrocyte specific genes. Wnt3A did not affect load-induced *col2a1 *transcription (Figure 3C), but inhibited load-induced *acan *mRNA expression after recovery (*P *< 0.001; Figure 3D), returning levels to those of untreated cells. The chondrocyte phenotype was maintained throughout culture as determined by *SOX9 *mRNA levels. Load induced *SOX9 *transcription following recovery (1.5-fold; *P *< 0.01, data not shown); however, this effect was abolished when cells were pre-treated with Wnt3A (data not shown).

### Divergence in the transcriptional regulation of catabolic enzymes in response to Wnt3A and tensile strain

**Figure 4:**
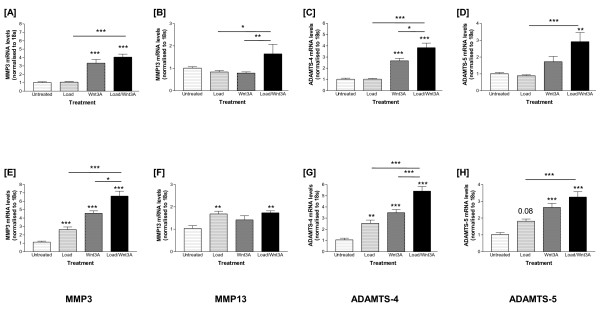
**Effects of tensile strain and Wnt3A, separately or combined, on catabolic proteinase transcription in chondrocytes**. Untreated chondrocytes served as controls. **(A) ***MMP3*, **(B) ***MMP13*, **(C) ***ADAMTS-4 *and **(D) ***ADAMTS-5 *gene expression levels analysed immediately or **(E)**, **(F)**, **(G) **and **(H) **following a four-hour recovery period respectively (refer to Figure 2 for calculations and statistical significance).

Transcript levels of key matrix metalloproteinases, *MMP3 *(Figure 4A, E), *MMP13 *(Figure 4B, F) and aggrecanases, *ADAMTS-4 *(Figure 4C, G), *ADAMTS-5 *(Figure 4D, H) were measured. Tensile strain did not affect proteinase transcription in cells analysed immediately. In contrast, Wnt3A stimulated *MMP-3 *(3.3-fold; *P *< 0.001, Figure 4A) and *ADAMTS-4 *transcription (2.7-fold; *P *< 0.001, Figure 4C), but did not affect *MMP13 *expression (Figure 4B). Differential transcriptional regulation of proteinases was observed when chondrocytes were stimulated with tensile strain and Wnt3A in combination. *MMP3 *transcription was induced immediately (relative to untreated four-fold; *P *< 0.0001 or tensile strain *P *< 0.001; Figure 4A). *MMP13 *transcription was also increased in co-stimulated cells (1.65-fold; *P *< 0.05 relative to strain or Wnt3A; Figure 4B). There was a synergistic induction of *ADAMTS-4 *mRNA levels (relative to untreated 3.6-fold: *P *< 0.001, tensile strain *P *< 0.001 or Wnt3A *P *< 0.05; Figure 4C). *ADAMTS-5 *transcription only increased when the stimuli were combined (relative to untreated 2.9-fold, *P *< 0.01 or tensile strain *P *< 0.001; Figure 4D).

Following recovery, tensile strain induced *MMP3 *(2.6-fold; *P *< 0.001, Figure 4E), *MMP13 *(1.7-fold; *P *< 0.01, Figure 4F) and *ADAMTS-4 *mRNA levels (2.5-fold; *P *< 0.01, Figure 4G), but *ADAMTS-5 *was not responsive (*P *= 0.08, Figure 4H). Wnt3A stimulated *MMP3 *(4.6-fold; *P *< 0.001; Figure 4F), *ADAMTS-4 *(3.3-fold: *P *< 0.001; Figure 4G) and *ADAMTS-5 *transcription (2.6-fold; *P *< 0.001; Figure 4H). Tensile strain and Wnt3A, in combination, induced *MMP3 *transcription (relative to untreated 6.6-fold; *P *< 0.001, tensile strain *P *< 0.001 or Wnt3A *P *< 0.05; Figure 4E). *MMP13 *transcription also increased in the co-stimulated cells (1.75-fold; *P *< 0.01), but levels did not exceed those observed in cells subjected to mechanical stimulation only (Figure 4F). Interestingly, there was an additive induction of *ADAMTS-4 *mRNA levels also (relative to untreated 5.4-fold; *P *< 0.001, tensile strain *P *< 0.001 or Wnt3A *P *< 0.001; Figure 4G). In comparison, there was a synergistic induction of *ADAMTS-5 *(3.3-fold, *P *< 0.001; Figure 4H). Although co-stimulation increased *MMP9 *transcription (1.4-fold; *P *< 0.01), mRNA expression returned to basal levels post-cessation of load (data not shown).

## Discussion

Articular cartilage functions to withstand mechanical load and provides a lubricating surface for frictionless movement of joints. However, cartilage degeneration can develop, leading to the progressive erosion of structural integrity and eventual loss of functional performance as characterised by the pathology of OA. Excessive or abnormal joint loading patterns can initiate OA cartilage pathology [3,12]. Further, a functional SNP resulting in an Arg324Gly substitution in sFRP3 (*FrzB*) has been identified as a susceptibility locus in association with hip OA [4]. The mutation in sFRP3 (*FrzB*) reduces its inhibitory activity *in vitro *[4], therefore, sustaining cellular Wnt signalling. A previously unasked question that we have sought to address in this study is whether mechanical load influences downstream responses mediated by canonical Wnt signalling in chondrocytes, that is, nuclear translocation of β-catenin, TCF/LEF transcriptional activation and induction of target genes, such as those encoding catabolic proteinases. This is of importance in trying to delineate the respective roles that both altered loading and genetic predisposition have in mediating cartilage degeneration.

Chondrocytes were pre-stimulated with recombinant Wnt3A for 24 hours prior to the application of tensile strain (7.5%, 1Hz) for 30 minutes. A peak strain of 7.5% at a frequency of 1 Hz was applied which is considered to be physiological [24,25]. Although chondrocytes predominantly experience compressive and shear forces *in vivo*, cells do encounter tensional forces particularly in the superficial zone [24]. Applying a physiological strain induced the transcription of type II collagen (*col2a1*), aggrecan (*acan*) and SOX9 (*SOX9*), which are all markers of the chondrocyte phenotype; the mechano-responsive nature of these matrix genes in chondrocytes has been previously reported [1,2]. Using this loading regime (in the absence of Wnt3A), β-catenin protein levels, as detected by Western blotting, were unaffected compared to expression in the untreated cells. The β-catenin antibody used in this study recognises both the phosphorylated and non-phosphorylated forms of the protein. However, using confocal microscopy, partial translocation of β-catenin to the nucleus was observed suggesting that this loading regime induces translocation rather than new synthesis; whether this is as a result of an alteration in phosphorylation status and/or a reduction in ubiquitination remains to be elucidated. This was a transient response because after the recovery period post-load, β-catenin was localised to the cytoplasm only. This is the first report of the mechano-responsiveness of β-catenin in chondrocytes, although mechanical activation of β-catenin signalling has been reported in the colon of APC^1638N/+ ^mice [26], mesenchymal stem cells [27] and periodontal ligament cells [28]. In contrast to the Wnt3A stimulated cells, where β-catenin is almost completely nuclear, an additive effect of load and Wnt3A on β-catenin localisation was observed with extensive β-catenin detected in the nucleus and throughout the cytoplasm. As discussed above, due to the nature of the β-catenin antibody we are unable to unequivocally state that the accumulation of β-catenin in the cytoplasm, in response to tensile strain and Wnt3A, is entirely the non-phosphorylated form. Clearly, there is an interaction between tensile strain and Wnt3A that promotes β-catenin translocation enhancing the activation of this pathway resulting in the additive/synergistic effects on downstream targets, for example, Lef1, MMPs. Interestingly, following the recovery period post-cessation of load the distribution of β-catenin differed in Wnt3A stimulated cells, either in the presence or absence of strain; two distinct populations of cells were noted. In cells stimulated with Wnt3A, a proportion of the cells contained not only nuclear β-catenin, as observed at the earlier time point, but also cytoplasmic labelling; whereas in Wnt3A stimulated cells subjected to tensile strain, a proportion of the cells contained almost exclusively nuclear β-catenin. This enhanced nuclear accumulation may explain why an additive induction of specific genes, for example, *c-fos, MMP3 *and *ADAMTS-4*, are observed in these cells post-recovery.

In the canonical Wnt/β-catenin pathway, binding of Wnt to the cell surface frizzled receptors leads to inhibition of β-catenin phosphorylation. Non-phosphorylated β-catenin translocates to the nucleus where it interacts with LEF/TCF transcription factors to activate Wnt target genes [29], which in chondrocytes include *c-myc*, *c-fos*, *c-jun *and *MMPs *[5-7,9,30]. In our study, *c-fos*, *c-jun*, *Lef1 *and the catabolic proteinases (*MMP3, ADAMTS-4 *and -*5*) were up-regulated in Wnt3A stimulated chondrocytes. When Wnt3A stimulated chondrocytes were exposed to tensile strain, if there was a response, one of three effects was observed: there was an additive effect, a synergistic induction or repression of gene expression. Load-induced expression of both *acan *and *SOX9 *was repressed in the presence of Wnt3A; Wnt3A-mediated SOX9 repression has previously been reported [30]. However, Wnt3A had no effect on load-induced *col2a1 *transcription. An additive effect was observed in up-regulating transcription of *c-fos*, *MMP3 *and *ADAMTS-4 *(particularly post-cessation of load), whereas there was a synergistic interplay of load and Wnt3A on *c-jun*, *Lef1 *and *ADAMTS-5 *transcription.

It is unknown how mechanical forces regulate canonical Wnt signalling in chondrocytes. However, increased β-catenin nuclear translocation was previously reported in chondrocytes subjected to low intensity pulsed ultrasound which was mediated via an integrin/phospatidyl-inositol-3-kinase/Akt signalling pathway [31]. In bone, strain-induced β-catenin translocation (2% strain, 3600 cycles) was shown to result from inhibition of GSK3β activity, which was suggested to be mediated via activation of the Akt pathway [32]. Several studies have indicated that signalling pathways, including the MAPK family [33], NFκB [34] and phosphatidyl-inositol-3-kinase/Akt [35] are activated by the Wnts in chondrocytes; these cascades are also activated in cartilage chondrocytes subjected to load [12,36,37]. Although it is currently unclear how these two pathways, mechanical stimulation and Wnt signalling, interact to co-ordinate the differential regulation of anabolic and catabolic genes in chondrocytes, we hypothesise that, as other studies have demonstrated [31,32], GSK3β activity is inhibited, which promotes β-catenin accumulation and nuclear translocation. One could speculate that both Wnt3A and mechanically-induced accumulation of β-catenin in the chondrocyte cytoplasm may be transmitted through reorganisation of the F-actin cytoskeleton given the known interactions of β-catenin with the cytoskeleton at the cell surface [38]. Our preliminary data demonstrate that Wnt3A stimulation can reorganise the F-actin network, from a predominantly cortical distribution to a more elaborate cytoplasmic network in primary chondrocytes (data not shown), as can mechanical load [39]. However, the upstream signalling cascades which may mediate these effects have yet to be unravelled and further experiments are required.

## Conclusions

Our data suggest that load and Wnt can interact to repress transcription of chondrocyte matrix/phenotype genes, whilst enhancing expression of catabolic mediators. This supports the finding that traumatic injury, initiated by cutting cartilage explants increased *Wnt16 *[40] and decreased *FrzB *mRNA expression; nuclear β-catenin translocation was also observed. Where aberrant Wnt signalling is evident, as occurs *in vivo *with the functional *FrzB *SNP, *FrzB*-/- or the *Col2a1-CreER^T2^; *β-*catenin^fx(Ex3)/wt ^*β-catenin cAct mouse, abnormal loading induced by joint misalignment, excessive load or traumatic injury could exacerbate downstream consequences of sustained Wnt signalling leading to a homeostatic imbalance of matrix catabolism over synthesis. Loss of the articular surface in weight-bearing regions in the synovial joints of the *Col2a1-CreER^T2^; *β-*catenin^fx(Ex3)/wt ^*(β-catenin cAct) mice indicates the importance of mechanical load, in conjunction with a susceptible genetic background as risk factors for cartilage pathology. Investigating how mechanical load (physiological and injurious) influences matrix homeostasis in these transgenic models *in vivo *will undoubtedly provide considerable insight on the interaction of load and genetics in OA predisposition. Understanding the cross-talk that exists between mechanical load and aberrant Wnt signalling may identify new molecular targets for the treatment of OA to effectively prevent structural damage of the cartilage to maintain its functional performance.

## Abbreviations

ADAMTS, a disintegrin and metalloproteinase with a thrombospondin type 1 motif; ECM, extracellular matrix; FITC, fluorescein isothiocyanate; *FrzB*, frizzled; ITS, insulin-transferrin-sodium selenite; MMP, matrix metalloproteinase; OA, osteoarthritis; sFRP3, secreted frizzled related protein 3

## Competing interests

The authors declare that they have no competing interests.

## Authors' contributions

RST contributed to the protein biochemistry studies, participated in the molecular biology experiments and performed the statistical analysis. ARC provided intellectual input to the conception and design of the study. VCD and EJB conceived of the study, and participated in its design and coordination. EJB conducted the protein biochemistry studies and molecular biology experiments, participated in the statistical analysis and drafted the manuscript. All authors critically appraised and approved the final manuscript.
